# Waldenström macroglobulinemia presenting as nephrotic syndrome: Renal heavy and light chain amyloidosis

**DOI:** 10.1002/jha2.424

**Published:** 2022-04-03

**Authors:** Arjan J. Kwakernaak, Sophie J. Bernelot Moens, Marc L. Hilhorst, Sandrine Florquin, Niels W.C.J. van de Donk, Josephine M.I. Vos

**Affiliations:** ^1^ Department of Internal Medicine Nephrology Clinical Immunology and Allergology Amsterdam University Medical Centers Amsterdam The Netherlands; ^2^ Department of Haematology Lymphoma and Myeloma Center Amsterdam (LYMMCARE) Amsterdam University Medical Centers Amsterdam The Netherlands; ^3^ Department of Nephrology Amsterdam University Medical Centers Amsterdam The Netherlands; ^4^ Department of Pathology Amsterdam University Medical Centers Amsterdam The Netherlands; ^5^ Department of Haematology Lymphoma and Myeloma Center Amsterdam (LYMMCARE) Amsterdam University Medical Centers Amsterdam The Netherlands; ^6^ Department of Haematology Lymphoma and Myeloma Center Amsterdam (LYMMCARE) Amsterdam University Medical Centers Amsterdam The Netherlands

**Keywords:** amyloidosis, M. Waldenström, nephrotic syndrome

A 71‐year‐old woman presented with nephrotic syndrome with preserved renal function. Immunofixation of the peripheral blood showed immunoglobulin M (IgM) *κ* paraproteinemia of 4.1 g/L with free *κ* light chain of 10.0 mg/dl and free *λ* light chain of 26.9 mg/dl, *κ*/*λ* ratio of 0.37 and differential free light chain (dFLC) of 16.9 mg/dl. Bone marrow biopsy demonstrated a B‐cell infiltrate with plasmacytoid differentiation, negative for CD5, CD23, and CD10, monoclonal for lambda, and presence of a *MYD88 L265P* mutation, consistent with Waldenström macroglobulinemia (WM). Congo red stain was negative. CT scan of the neck/thorax/abdomen showed neither lymphadenopathy nor organomegaly. Kidney biopsy was performed and showed subtle enhancement of mesangial areas by light microscopy (Figure 1A), suggestive of depositions, with immunofluorescence demonstrating IgM heavy chain as well as λ light chain deposition (Figure 1C,D). Electron microscopy showed fibrils with a thickness of 9 nm consistent with amyloid deposition (Figure 1B). Congo red staining was only slightly positive indicating a modest amount of amyloid. We diagnosed this patient with WM and renal heavy and light chain amyloidosis. There was no macroglossia, periorbital purpura, neuropathy or autonomic dysfunction. Furthermore, there were no signs of concurrent cardiac involvement. We started treatment with rituximab (R)‐bortezomib‐dexamethasone. After one cycle, treatment was switched to R‐bendamustine because of treatment‐emergent grade 2 neuropathy. The nephrotic syndrome was additionally treated with furosemide, spironolactone, hydrochlorothiazide, a statin and anticoagulation. After the first cycle of R‐bendamustine, she reached renal response (reduction of 24‐h protein excretion from 11.6 to 0.13 g/day) and a very good partial hematological response (VGPR: lowering of dFLC to < 4 mg/dl).

**FIGURE 1:**
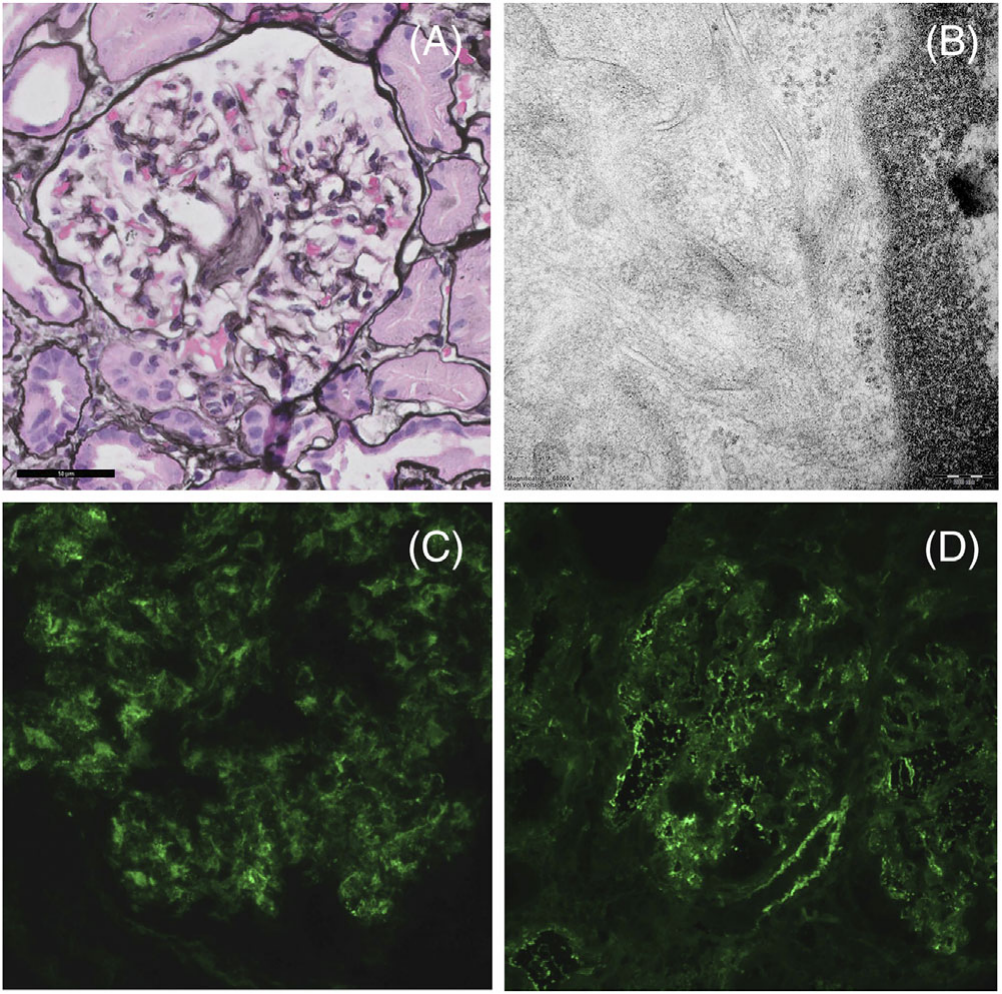
(A): Light microscopy with Jones silver‐stained (x40); (B): Electron microscopy; (C): Immunofluorescence with IgM staining; (D): Immunofluorescence with lambda light chain enhancement (Immunofluorescence for kappa, IgG, IgA and complement was negative)

Kidney involvement in WM is rare (estimated prevalence 5%) and presents with a wide range of nephropathology. AL amyloidosis or monoclonal immunoglobulin deposition disease due to heavy or light chain deposition are well‐known causes of nephrotic syndrome in the context WM or IgM monoclonal gammopathy of unknown significance. Light and heavy chain amyloidosis (AHL amyloidosis), however, is rare, and in the context of IgM paraproteinemia/WM less than five cases have been published. The clinical presentation of patients with renal AHL amyloidosis deviates from those with conventional AL amyloidosis: cardiac involvement is less likely, fat pad and bone marrow biopsy are typically negative for amyloid, and a circulating complete monoclonal immunoglobulin is more frequently found. These differences highlight the necessity for kidney biopsy to confirm cases of renal AHL. This confirmation may be of important prognostic value: patients with renal AHL seem to have better renal response to therapy and better overall survival compared to those with conventional renal AL amyloidosis.

## CONFLICT OF INTEREST

The authors declare no conflict of interest.

## FUNDING INFORMATION

The authors received no specific funding for this work.

## ETHICS STATEMENT

We comply to practice guidelines on research integrity and publishing ethics and the committee on publication ethics. Patient provided written consent for publication of this case.

## AUTHOR CONTRIBUTIONS

A.J.K. and S.J.B.M. wrote the manuscript with N.W.J.D. and J.V. M.L.H. performed the kidney biopsy and provided considerate intellectual contribution to the manuscript. S.F. evaluated the kidney biopsy specimen and provided the figure.

## Data Availability

Data available on request from the authors.

